# Tumour Cell Membrane Poration and Ablation by Pulsed Low-Intensity Electric Field with Carbon Nanotubes

**DOI:** 10.3390/ijms16046890

**Published:** 2015-03-26

**Authors:** Lijun Wang, Dun Liu, Ru Zhou, Zhigang Wang, Alfred Cuschieri

**Affiliations:** Institute for Medical Science and Technology (IMSaT), College of Medicine, Dentistry and Nursing, University of Dundee, Dundee DD2 1FD, UK; E-Mails: l.y.wang@dundee.ac.uk (L.W.); dun.d.liu@gmail.com (D.L.); rachelqiaoqiao@gmail.com (R.Z.); a.cuschieri@dundee.ac.uk (A.C.)

**Keywords:** electrical pulse, electroporation, tumour cell ablation, carbon nanotubes, cell membrane permeabilization

## Abstract

Electroporation is a physical method to increase permeabilization of cell membrane by electrical pulses. Carbon nanotubes (CNTs) can potentially act like “lighting rods” or exhibit direct physical force on cell membrane under alternating electromagnetic fields thus reducing the required field strength. A cell poration/ablation system was built for exploring these effects of CNTs in which two-electrode sets were constructed and two perpendicular electric fields could be generated sequentially. By applying this system to breast cancer cells in the presence of multi-walled CNTs (MWCNTs), the effective pulse amplitude was reduced to 50 V/cm (main field)/15 V/cm (alignment field) at the optimized pulse frequency (5 Hz) of 500 pulses. Under these conditions instant cell membrane permeabilization was increased to 38.62%, 2.77-fold higher than that without CNTs. Moreover, we also observed irreversible electroporation occurred under these conditions, such that only 39.23% of the cells were viable 24 h post treatment, in contrast to 87.01% cell viability without presence of CNTs. These results indicate that CNT-enhanced electroporation has the potential for tumour cell ablation by significantly lower electric fields than that in conventional electroporation therapy thus avoiding potential risks associated with the use of high intensity electric pulses.

## 1. Introduction

Electric fields have a range of effects on cells and tissues. Low intensity (tens of mV/cm to a few V/cm) electric field can affect cell division, polarization and migration [1,2,3]; whereas cell plasma membrane is permeabilized when the electric field is sufficiently high (above a few hundred V/cm to one kV/cm), a process called electroporation [4,5,6]. As a traditional physical method to increase permeabilization of plasma membrane of the cells, electroporation has been explored widely in many biomedical applications such as gene transfection and drug delivery. Clinically, the application of short and high-voltage pulses to cause disorganization of the lipid structure and thereby enhance the delivery of pharmaceutical molecules is referred to as electroporation. In cancer treatment, use of a locally applied electrical field to enhance cell permeability thus permitting intracellular accumulation of cytotoxic anticancer drug is known as electrochemotherapy. The extent of cell membrane permeabilization is determined by several factors including field amplitude, duration and pulse frequency, and can be reversible or irreversible. In any case, the requirement of high voltage, especially in irreversible electroporation of cancer ablation (usually a few kV/cm), may create serious problems such as cardiac arrhythmias and uncontrolled muscle contractions [7,8]. Nanosecond pulsed electric fields, which create pores not only in plasma membranes but also in intracellular membranes, have also been exploited recently for cancer treatment [9]. These ultra short pulses induce cell death primarily by triggering apoptosis process, and require even higher voltage or field (to tens of kV/cm). Recent advances in nanotechnology have shed some light to overcome these limitations. Use of carbon nanotubes (CNTs) to enhance the electric field and to reduce the required voltage for effective electroporation or tumour ablation, is one of these options and early studies with this approach have been promising [10,11]. 

CNTs are tiny, seamless tubes with hexagonal lattices formed by rolling single (single-walled, SWCNT) or multiple layers (multi-walled, MWCNT) of graphite sheets [12,13,14]. The most predominant structural feature of CNTs is the large length to diameter ratio (L/D, aspect ratio) of up to 1000, so they are usually regarded as nearly one-dimensional structures [15]. CNTs have attracted much attention in recent years owning to their unique mechanical and electrical properties. Depending on chirality and other factors, CNTs can perform as a metallic or a semiconducting material [16]. In terms of electric polarizability, it has been reported that the responses of CNTs to an external electric field strongly depends on their electronic structure when electric field is applied parallel to the cylindrical axis on the CNT; whereas the tube diameter was dominant when electric field was applied perpendicular to the cylindrical axis [17,18]. It has been reported that MWCNTs can enhance the applied electric field via their “lightning rod” effect [10,19], *i.e*., amplifying the electric field. The field enhancement at the tips of MWCNTs can be estimated by the following Equation (1).

(1) $$\frac{E}{E_{0}}=\text{α}\frac{L}{D}$$

where *E* is the field strength at the tip of CNT, *E*_0_ is the applied external field strength, α is a constant, *L* is the CNT length and *D* is the diameter. The high aspect ratio (*L*/*D*) of CNTs explains the reason why CNTs are excellent at concentrating field, thereby facilitating the electroporation. This phenomenon indicates the production of localized electroporation with relatively low field strength [10,19]. In the study by Rojas-Chapana *et al*., MWCNTs were used for microwave electroporation of gram-negative bacteria. They reported that MWCNTs could strongly enhance the electric field strength at their ends, creating pores in the membrane, thereby allowing the uptake of exogenous substances into cells [19]. An experimental study [11] was carried out using a lab-on-a-chip device containing electrodes with or without deposited CNTs in which cell suspensions were passed while voltages of various amplitudes were delivered. The results obtained showed that with the CNT-deposited electrodes, the voltage required to achieve >95% cell lysis was only 35 V/cm, compared with 135 V/cm for the CNT-free electrodes. The authors ascribed this difference to the field enhancement effect by the CNTs. Based on the different transverse and longitudinal dielectric properties of MWCNTs, Raffa *et al*. built a novel CNT-based electroporation system that consisted of two pairs of electrodes placed at right angle to each other. Their results showed that with this CNT-enhanced method the electric field strength required for reversible cell electroporation was dramatically reduced [10]. In addition to the field enhancing effect, CNTs appeared to be also able to deform cell membrane by direct physical contact. Our simulation study has suggested that electromagnetic field-induced dielectrophoretic force on CNT tips was sufficient to cause cell membrane deformation and poration [18]. Experimentally, CNTs were shown to induce cell permeabilization in alternating magnetic field, further implicating the link between physical contact and force by CNTs with membrane disruption [20]. The potential dual effects, *i.e*., the local field enhancing and physical disrupting ability, of CNTs on cell membrane under alternating electromagnetic fields prompted us to explore the use of low intensity electrical pulses to permeablize cell membrane or even ablate cancer cells in the presence of CNTs.

Reversible cell membrane permeabilization, in which electroporated membrane reseals after electrical pulse application and most cells recover from the treatment and remain viable, has been extensively researched for its potential in nucleic acid transfection, gene and drug delivery and *etc*., and has been applied clinically for cancer treatment [21,22]. The use of electrical pulses in irreversible cell damage and tissue ablation, on the other hand, has been explored more recently [7,8]. When the electrical fields cause permanent permeabilization of the membrane and consequent loss of cell homeostasis, irreversible electroporation, *i.e*., cell death (primarily necrosis) occurs. In recent years, irreversible electroporation has been gaining interest as an emerging medical technology for minimally invasive treatment of cancer [8,23]. Like the use of electrical pulses for many other applications, the level of voltage is always a major concern in electroporation and electrical ablation. In order to investigate how the electric field intensity can be reduced by CNTs to achieve reversible or irreversible cell membrane permeabilization, we developed a custom-designed electrical pulse generating system in which two electric fields, one perpendicular with another, are operated in an alternating manner. Using this system we studied optimal electrical parameters, under which both cell membrane permeabilization and cell viability are taken into account, for CNT-enhanced effective electroporation. By selection of the optimized parameters for the main and alignment electric fields in our experiments, we were able to dramatically reduce the voltage needed to induce significant cell membrane poration as well as irreversible cell damage in the presence of CNTs. The observed effects by CNTs may have potential applications in irreversible electroporation for tumour ablation or in combination with cytotoxic drug for electrochemotherapy by low intensity electrical fields.

## 2. Results and Discussion

### 2.1. The Established Electroporation Apparatus

**Figure 1:**
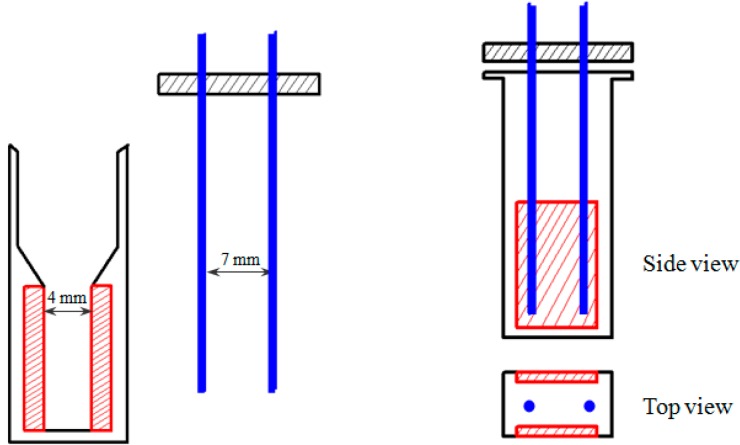
Schematic representation (not drawn to scale) of the electroporation cuvette assembled with two pairs of electrodes: main electrodes (red) and alignment electrodes (blue).

Our custom-designed electroporation system consisted of electroporation circuit, power supply, signal generator, voltage amplifier, the main electrodes (two plate electrodes assembled in a standard electroporation cuvette with 4 mm gap between the electrodes) and the alignment electrodes (two needle electrodes). The two pairs of electrodes were perpendicular; the main electrodes with 4 mm gap were commercially equipped within the standard electroporation cuvette while the needle electrodes with 7 mm gap made from stainless steel were used for CNTs alignment (Figure 1).

The required voltages for the main and alignment pulses were realized by amplification of the signals from the signal generator by a commercial high voltage amplifier and a LM3886 power amplifier circuit, respectively. Upon circuit test (for the alignment pulses), calibration of the system and measurement of the signal outputs, the main and alignment pulses (E_M_ and E_A_, respectively) at a maximum field strength of 60 and 30 V/cm was achieved. We considered that these voltages would be sufficient for the required electric field intensity for the MWCNT-assisted cell membrane permeabilization.

### 2.2. Effect of MWCNTs on Cell Membrane Permeability by the Two Electric Fields

**Figure 2:**
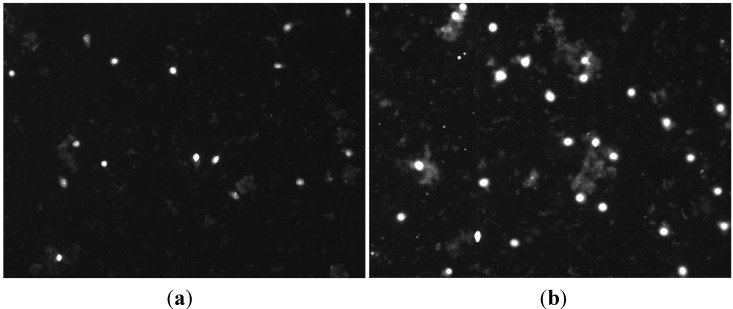
Fluorescence micrograph of propidium iodide (PI) cellular uptake in MCF-7 cells by electroporation using 50 pulses and frequency 1 Hz in the absence (**a**) and presence (**b**) of multi-walled carbon nanotubes (MWCNTs) (30 μg/mL). The field intensity of main pulses (E_M_) was 20 V/cm and the alignment field (E_A_) intensity was 15 V/cm. Cell suspension samples in the same density (10^6^ cells/mL) were treated as described above and stained with PI and viewed under microscope as described in the Experimental Section. As cell density was identical in both samples, only fluorescence images were shown here.

We first tested the field enhancing effect of MWCNTs on membrane permeabilization by using an electric field intensity of the main pulses as low as 20 V/cm and alignment pulses of 15 V/cm. Propidium iodide (PI) is a membrane impermeant fluorescent molecule and generally excluded from viable cells with structurally normal membrane. PI is widely used in cell poration assessment as membrane poration allows the dye to pass into and fluorescently stain the cells. As observed by fluorescence microscopy, the presence of MWCNTs in the pulsing media clearly increased the PI cellular uptake even at such low field intensity; the number of PI-positive cells was approximately one fold higher than in no-MWCNT controls (Figure 2). These initial data indicate that the MWCNTs can help to reduce the electroporation voltage required for effective cell membrane poration. We therefore carried further experiments to quantify the enhancement by the MWCNTs under a variety of conditions with our established electroporation system by focusing on the field intensity, pulse number, and pulse frequency. For these experiments, we used Trypan Blue (TB), another membrane impermeant dye commonly used for staining permeabilized cells, in order to count the stained cells immediately after pulse application.

### 2.3. Effect of the Field Strength on Cell Membrane Poration Enhanced by the MWCNT

**Figure 3:**
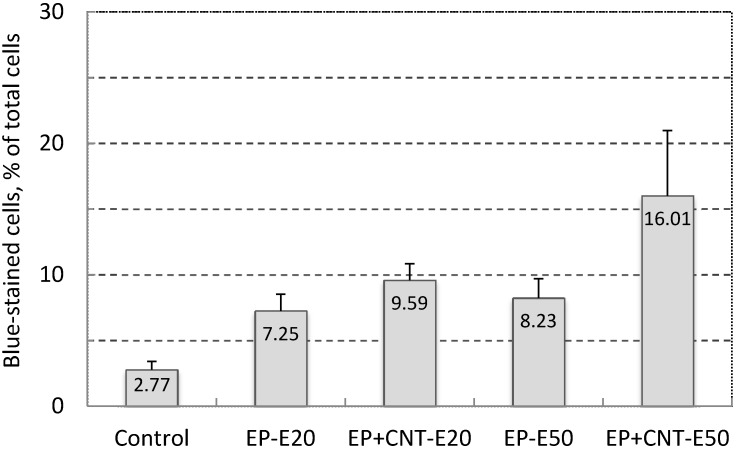
Effect of field strength on cell permeability (Trypan Blue-stained cells, in % of total cells) of MCF-7 cells by electroporation, using 100 pulses per treatment at 1 Hz. Control: cells without any treatment; EP-E20/EP-E50: cells were pulsed with E_M_ 20 V/cm and E_A_ 15 V/cm, E_M_ 50 V/cm and E_A_ 15 V/cm; EP + CNT-E20/EP + CNT-E50: cells were treated with the parameters described above in the presence of MWCNTs (30 μg/mL).

As shown in Figure 3, when we treated the cells under two field intensities (20 and 50 V/cm) with other pulsing parameters being kept constant, there was a general trend that increasing the field strength further increased the electroporation efficiency demonstrated by the higher electro-induced permeabilization in the treated groups by pulsation of 50 V/cm from the main electrodes (EP + CNT-E50) than by pulses of 20 V/cm (EP + CNT-E20). In addition, more cells were stained by the TB in the experimental groups in which MWCNTs were present (EP + CNT-E20 and EP + CNT-E50, respectively) than that in the corresponding groups with no MWCNTs. In particular, at the field intensity 50 V/cm of the main field, the increase in the percentage of permeabilized cells in the presence MWCNTs was significant (*p* < 0.05). Therefore, increasing the field intensity as with conventional electroporation (using the main electrodes only), result in higher electroporation and hence cell permeabilization [4]. In order to optimize the MWCNT-mediated electroporation whilst still to keep the pulsing voltage as low as possible, we fixed the main and alignment pulses at 50 and 15 V/cm, respectively, while varying other parameters in the following electroporation experiments.

### 2.4. Influence of Pulse Number and Frequency on MWCNT-Enhanced Cell Membrane Poration

Pulse number and frequency have been implicated to be important factors in electrical pulse induced cell membrane permeabilization [5,24]. We observed that when the cell suspensions were submitted to varied numbers of pulses of E_M_ = 50 V/cm and E_A_ = 15 V/cm at 1 Hz, there was a trend towards an increase in the blue-stained cells with increasing number of pulses (Table 1). This may be partially due to an increased degree of instability of cell membrane with the increasing number of pulses [5]. Again the comparison between the treatments with and without MWCNTs showed greater numbers of permeabilized (blue-stained) cells in the treatment groups with MWCNTs than their counterparts. When the number of pulses was increased to 500, the percentage of blue-stained cells reached 23.03%, 1.88-fold higher than that in the absence of MWCNTs (*p* < 0.05).

In the presence of MWCNTs, the yield of electroporated cells increased from 23.03% to 38.62% when the frequency of applied 500 pulses was increased from 1 to 5 Hz during the pulsation (Table 1, *p* < 0.05). The frequency-dependence could be attributed to the reported observation that pulse energy can be delivered to the same area within defined short period of time with increased pulses frequency [24]. Moreover, the enhancement by MWCNTs in these treatments was maximized when the 500 pulses were applied at frequency of 5 Hz: a 2.77-fold increase was observed by the comparison of cell permeabilization rate from the 500 pulses/5 Hz treatment groups in the presence and absence of MWCNTs (Table 1). These data reveal that both the pulse number and frequency are important factors in determining the efficiency of cell membrane permeabilization by MWCNT-enhanced electroporation. As further increases in either the pulse number or the frequency caused problems such as cell/dye aggregation and difficulties in cell counting (data not shown) due to potential electro-chemical effects, we consider the obtained parameters in our experimental system (E_M_ = 50 V/cm, E_A_ = 15 V/cm, pulse number = 500, pulse frequency = 5 Hz) as providing the optimal conditions. We then further studied cell viability and cellular drug uptake with these parameters.

**Table 1:** Efficiency of cell membrane permeabilization (%) of MCF-7 cells by the applied electrical pulses of varying number and frequency in the absence and presence of MWCNTs (30 μg/mL). The applied field strength was kept at E_M_ 50 V/cm and E_A_ 15 V/cm and the percentage of blue-stained cells was counted and calculated upon the application of various electrical pulse parameters as described in the Materials and Methods. EP: cells were pulsed with the indicated pulse frequency and number; EP + CNT: cells were treated with the indicated parameters in the presence of MWCNTs (30 μg/mL). Significant differences in the percentage of membrane permeabilization were observed between experimental groups under either EP or EP + CNT conditions, respectively. *, ^#^, and ** indicate *p* < 0.05.

Electroporation Condition	Pulse Frequency (Hz)			
	1			5
Pulse number	100	200	500	500
EP	7.46 ± 0.91 *	13.61 ± 8.37	12.22 ± 2.28 *	13.92 ± 1.61
EP + CNT	12.20 ± 6.36 *	16.73 ± 0.68 ^#^	23.03 ± 2.84 *^,#,^**	38.62 ± 11.30 **
Fold of EP + CNT/EP	1.64	1.23	1.88	2.77

### 2.5. Cell Viability upon Application of the Optimized Electric Field Parameters in the Absence and Presence of a Cytotoxic Drug

**Figure 4:**
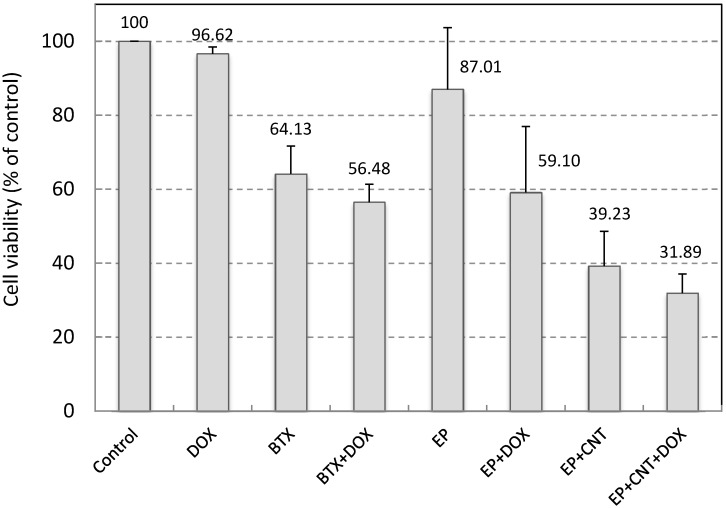
Cell viability of MCF-7 cells by electroporation. 500 pulses at 5 Hz with E_M_ 50 V/cm, E_A_ 15 V/cm were applied. Control: cells without any treatment; DOX: addition of 0.05 μM Doxorubicin in the media without electroporation; BTX (EP)/BTX + DOX (EP + DOX): cells suspended in the pulsing media without or with 0.05 μM Doxorubicin; EP + CNT/EP + CNT + DOX: cells suspended in the pulsing media containing 30 μg/mL MWCNTs in the absence or presence of 0.05 μM Doxorubicin. Doxorubicin in culture media was diluted by 100× after electroporation (to 0.5 nM) and cells were further incubated for 24 h prior to the cell viability assay.

We used Doxorubicin (DOX) to examine the potential of the applied electrical pulses plus MWCNTs on cell drug uptake and electrochemotherapy. DOX was used at very low concentration (0.05 μM in the original pulsing media and 0.5 nM by further dilution during cell culture post electroporation). At this concentration, DOX had little effect on cell viability in MCF-7 cells after 24 h exposure (Figure 4). A conventional electroporation procedure, using a standard system (BTX electroporation system) and a single 750 V/cm pulse (10 ms), was employed as a reference for the comparison of the outcome. Electroporation at 750 V/cm using the standard system induced a significant reduction in cell viability (*p* < 0.05) and a very small enhancement in cytotoxicity by DOX (Figure 4). Application of the optimized electrical parameters alone, on the other hand, slightly but not significantly (*p* > 0.05) reduced cell viability (87.01% ± 16.70% of control), indicating that use of these pulse parameters did not cause much damage to the cells in the absence of MWCNTs. When MWCNTs were supplied in the pulsing media, the utilization of our custom-designed electroporation system induced a large decrease in cell viability compared to that without MWCNTs (39.23% *versus* 87.01%, *p* < 0.05, Figure 4). In addition, we have shown in our previous study that when incubated with MCF-7 cells for 24 h at this concentration, MWCNTs did not elicit significant cytotoxicity [25]. Therefore, these results indicate that the MWCNTs-amplified electric field under these conditions resulted in largely irreversible cell electroporation. Thus, the low cell viabilities (31.89%) in the DOX exposure group under the same conditions could be attributed to the enhanced cellular intake of DOX as well as damage to cells caused by the irreversible cell membrane poration. Data for the two drug exposure groups, EP + DOX and EP + CNT + DOX, showed significantly different level of viable cells post treatments (59.10% *versus* 31.89%, *p* < 0.05). However, the low cell viability in the absence of DOX and presence of MWCNTs (EP + CNT), compared to drug exposure group under the same pulsing conditions (EP + CNT + DOX) (39.23% *versus* 31.89%, *p* > 0.05), suggested that MWCNT-enhanced irreversible electroporation might play a major role by directly killing the tumour cells. Further experiments using DOX at clinically relevant concentrations are needed to verify the effects on cellular drug uptake by MWCNT-amplified electrical fields, which may include potential synergistic impact from MWCNTs (physical contact). Overall, the MWCNT-enhanced cell destruction by low intensity electric fields indicates the potential of combining CNTs with an appropriated electroporation system for effective electroporation and warrants further investigation.

## 3. Experimental Section

### 3.1. Custom-Designed Electroporation System

**Figure 5:**
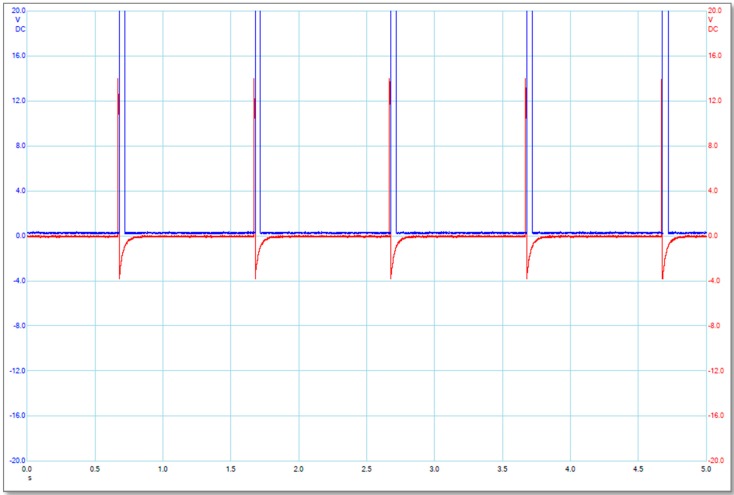
Illustration of the electrical pulse signals produced by the main electrodes (blue or larger ones) and the alignment electrodes (red or smaller ones). The label for *X*-axis is Time in second scale (s), and the label for *Y*-axis is Applied Voltage in Volt (V).

The two pulse waves were initially generated by a two-channel arbitrary signal generator (TGA 1243 Arbitrary Waveform Generator, A&T Thandar Instrument, Huntingdon, UK), and then were separately amplified by a high voltage amplifier (A-303 High Voltage Amplifier and Modulator, A.A. Lab-Systems, Ramat-Gan, Israel) and a custom-designed power amplifier(LM3886, National Semiconductor, Santa Clara, CA, USA). A Power Supply (Stabilised Power Supply, Farnell, Leeds, UK and EL561R Power Supply, A&T Thandar Instrument, Huntingdon, UK) provides stable DC power to the LM3886 power amplifier. A Picoscope (PICO Technology, St Neots, Cambridgeshire, UK) was used to measure and record electrical signal data. The main pulses (M) and alignment pulses (A) were applied to the pulsing media by the main electrodes and alignment electrodes, respectively. The alignment pulses were used to align the MWCNTs in order to obtain the maximum amplification of the external electric field [10,11]. The main and alignment pulses were synchronized (each main pulse starts at the end of the alignment pulse) as shown in Figure 5.

### 3.2. Cell Culture and Cell Electroporation

The cell line used for this study was human breast cancer cells MCF-7 (ATCC, Teddington, UK; Cat# CCL-228). MCF-7 cells were grown in DMEM (GIBCO, Invitrogen, Paisley, UK). Media were supplemented with 10% fetal calf serum, 2 mM glutamine, 100 IU/mL penicillin, and 100 μg/mL streptomycin. Cells were grown under standard cell culture conditions in 5% CO_2_ at 37 °C to reach confluence of 60%–70% before subjected to any further treatment. MCF-7 cells cultured in 75 cm^2^ flasks were trypsinized and suspended in the pulsing media at a concentration of 10^7^ cells/mL for electroporation. The pulsing media consisted of OPTI-MEM (Gibco, Paisley, UK; Cat# 11058-021) with or without 30 μg/mL of MWCNTs (Nanothinx S.A, Patras, Greece) coated with a non-ionic surfactant Pluronic F-127 (Sigma Aldrich, Poole, UK; Cat# P2443) [25]. 400 μL cell suspension mixed with 80 μL Trypan Blue (TB; 0.4%, *v*/*w*) or 2 μL propidium iodide (PI, Abcam, Cambridge, UK; Cat# ab14085) was placed in the electroporation cuvette. The pulse intensity of the main field (E_M_) of 50 or 20 V/cm, and the alignment pulses (E_A_) of 15 V/cm was applied. The pulse durations of the main and alignment pulses were fixed at 40 and 10 ms, respectively. The number of pulses varied from 50 to 500 and the frequencies used were 1 or 5 Hz.

Some experiments were also carried out by using a commercial electroporation apparatus (ECM 830 Square Wave Electroporation System, BTX Harvard Apparatus, Holliston, MA, USA) for comparison purpose, using parameters as essentially described by the manufacturer for MCF-7 cells, *i.e*., a single pulse of 750 V/cm for 10 ms was applied to the cell suspension.

### 3.3. Analysis of Cell Membrane Permeabilization

The efficiency of electroporation was first evaluated by the penetration of nonpermeant dyes, TB and PI across the cell membrane. After electroporation, the treated cells were incubated at room temperature for 10–15 min (protected from light when PI was applied) before examined under the bright field (for TB) or fluorescence microscope (for PI). Photos were taken with more than three 10× microscopic fields. For TB staining, numbers of stained and total cells were counted in each photo under grids, and only the microscopic fields with 300–2000 cells were taken into account. The percentage of permeabilized cells was counted as the ratio of the number of stained cells to the total cell number. Experiments were repeated for four times, and data were expressed as means ± SD.

### 3.4. Evaluation of Cell Viability upon Electrical Pulse Application

The effect of electroporation was also assessed by cell viability using CellTiter-Blue Cell Viability Assay (Promega, Southampton, UK). In this assay, viable cells convert a non-fluorescent compound resazurin into fluorescent end product resorufin and the cell viability was quantitatively measured by fluorescence intensity. After electroporation cells were seeded in 96-well plates (10^4^ cells in each well) and incubated under standard cell culture conditions over 24 h prior to the viability assay and fluorescence intensity (560_ex_nm/590_em_nm) was measured by a microplate reader (TECAN, Grödig, Austria). Values of treated cells were expressed as percentage of that from corresponding control cells. In some experiments, Doxorubicin was present in the media during electroporation for assessing the enhanced drug uptake upon membrane permeabilization. Experiments were repeated for four times and data were expressed as means ± SD.

## 4. Conclusions

Optimized pulse parameters were investigated in our custom-designed perpendicular electric field system to evaluate the MWCNT-enhanced electroporation of cancer cells *in vitro*. Our data demonstrated that MWCNTs can significantly increase low intensity electric field-induced plasma membrane permeabilization in tumour cells by approximately three fold. We also observed that MWCNT-enhanced irreversible electroporation caused predominantly the reduction of cell viability in the pulsed cells. We conclude that the combination of an appropriate electrical pulsing system with CNTs should be investigated further with regard to various factors involved in the effective irreversible electroporation including the enhancement of the electric fields by CNTs, the direct physical contact of CNTs with cell membrane and potential Joule effect. We also recognize that in future both in *in-vitro* experiments using a wider range of cell lines and in *in-vivo* studies should be included to explore effective cancer treatment and tumour ablation with reduced risks encountered in conventional high voltage electric field.
